# Metabolic Reprogramming of Macrophages upon In Vitro Incubation with Aluminum-Based Adjuvant

**DOI:** 10.3390/ijms24054409

**Published:** 2023-02-23

**Authors:** Ravi Danielsson, Nathan Ferey, Irene Mile, Håkan Eriksson

**Affiliations:** 1 Department of Biomedical Science, Faculty of Health and Society, Malmö University, 205 06 Malmö, Sweden; 2 Polytech Clermont-Ferrand, Universite Clermont Auvergne, 63001 Clermont-Ferrand, France

**Keywords:** aluminum adjuvant, macrophages, metabolic reprogramming, lactate

## Abstract

Aluminum-based adjuvants have been extensively used in vaccines. Despite their widespread use, the mechanism behind the immune stimulation properties of these adjuvants is not fully understood. Needless to say, extending the knowledge of the immune-stimulating properties of aluminum-based adjuvants is of utmost importance in the development of new, safer, and efficient vaccines. To further our knowledge of the mode of action of aluminum-based adjuvants, the prospect of metabolic reprogramming of macrophages upon phagocytosis of aluminum-based adjuvants was investigated. Macrophages were differentiated and polarized in vitro from human peripheral monocytes and incubated with the aluminum-based adjuvant Alhydrogel^®^. Polarization was verified by the expression of CD markers and cytokine production. In order to recognize adjuvant-derived reprogramming, macrophages were incubated with Alhydrogel^®^ or particles of polystyrene as control, and the cellular lactate content was analyzed using a bioluminescent assay. Quiescent M0 macrophages, as well as alternatively activated M2 macrophages, exhibited increased glycolytic metabolism upon exposure to aluminum-based adjuvants, indicating a metabolic reprogramming of the cells. Phagocytosis of aluminous adjuvants could result in an intracellular depot of aluminum ions, which may induce or support a metabolic reprogramming of the macrophages. The resulting increase in inflammatory macrophages could thus prove to be an important factor in the immune-stimulating properties of aluminum-based adjuvants.

## 1. Introduction

Almost 100 years ago, Glenny et al. [1] introduced aluminous compounds as adjuvants in vaccine preparation. Aluminum-based adjuvants (ABAs) have been extensively used, but the mechanisms behind the immune-stimulating properties of ABAs are yet not fully comprehended. It is commonly recognized that upon administration, ABAs enhance the antigen uptake by antigen-presenting cells (APCs) and induce inflammation [2]. To the best of our knowledge, on the protein level, only inflammatory cytokines of the IL-1 family are induced by ABAs in in vitro studies [3,4,5]. However, the induction of IL-1β is not crucial for the immune-stimulating properties of ABAs in vivo [6,7]. Hence, other mechanisms must exist that mediate the immune-stimulating properties of ABAs. These mechanisms need to be identified to aid in our efforts to create safe and effective vaccines.

Dendritic cells (DCs) and macrophages are important sentinel cells of the innate immune system. Macrophages are commonly referred to as quiescent M0 macrophages, inflammatory M1 macrophages, and alternatively activated M2 macrophages [8,9]. M1 macrophages have pro-inflammatory functions, whereas M2 macrophages, also referred to as anti-inflammatory macrophages, are involved in wound healing and in damping of the immune response [10]. Polarized macrophages have altered metabolic pathways, and their metabolism is correlated to their biological function [11]. Inflammatory M1 macrophages and activated DCs have a metabolic signature of enhanced glycolytic metabolism and decreased oxygen consumption [12,13] whereas the metabolism of M2 macrophages is characterized by oxidative phosphorylation and fatty acid oxidation [14].

Phagocytosis of particles increases the energy demand of phagocytosing cells, and thus it is essential to investigate if ABAs provoke any changes to the metabolic signatures of macrophages. In this paper, in vitro polarized macrophages were incubated with the Alhydrogel^®^, followed by an analysis of the cellular content of lactate, a marker of increased glycolytic metabolism.

## 2. Results

**Figure 1:**
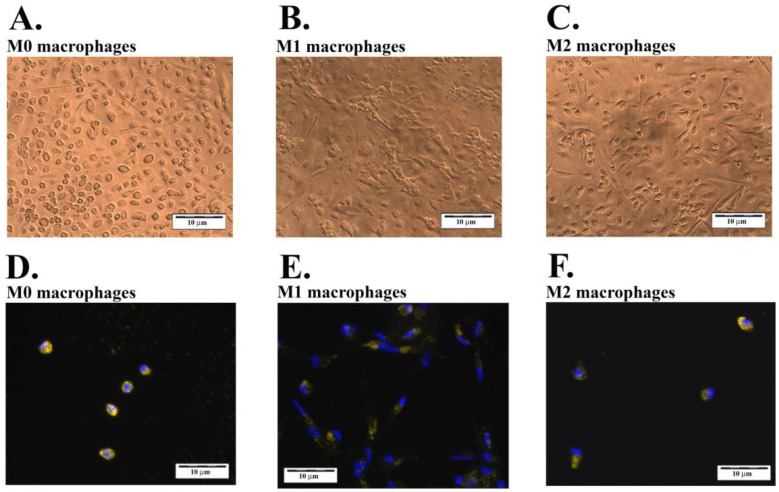
Phase contrast (**A**–**C**) and fluorescence (**D**–**F**) microscopy images of differentiated and polarized macrophages at 200× magnification. (**A**,**D**): quiescent M0 macrophages; (**B**,**E**): inflammatory M1 macrophages; (**C**,**F**): alternatively activated M2 macrophages.

To explore the effect of ABAs on the metabolism of macrophages, human peripheral blood monocytes were differentiated in vitro, forming M0 macrophages that were further polarized to M1 and M2 macrophages. In Figure 1, phase contrast microscopy images of the macrophages show the dendritic shape of the M1 macrophages (Figure 1B) and the round to oval shapes and the almost needle-shaped morphology of the M2 macrophages (Figure 1C), the latter of which is similar to the morphology of the quiescent M0 macrophages (Figure 1A). Upon double staining with the blue fluorescent nucleus stain DAPI, to identify the cells, and the green fluorescent lumogallion labeled Alhydrogel^®^, the macrophages became associated with Alhydrogel^®^ (Figure 1D–F). As judged by the fluorescence microscope images and adjusting the focal plane, all macrophage varieties were capable of phagocytosing ABAs (Figure 1D–F), All examined macrophages contained several particles after incubation with Alhydrogel; however, no attempt was made to quantitate and compare the phagocytosing capacity across the three macrophage forms.

After differentiation and polarization at day 5, conditioned media from differentiated and polarized macrophages were analyzed regarding their cytokine content by ELISA. Macrophages were detached from the culture wells, stained with antibodies against CD markers and analyzed by flow cytometry. All M1 macrophages clearly expressed CD80, whereas the M0 and M2 macrophages showed low or almost no expression of CD80. All macrophages expressed CD206, and upon polarization, the expression of CD206 of M1 macrophages was downregulated compared to the M0 macrophages, whereas the M2 macrophages displayed a competent upregulation of CD206. The M1 macrophages secreted high amounts of both TNF-α and IL-6 into the culture medium, often out of range of the ELISA calibration curve. On the other hand, TNF-α and IL-6 were just detectable in the culture medium from M0 and M2 macrophages, and often the concentrations fell below the ELISA detection limit. A summary of the expression of surface markers and cytokine production by the differentiated and polarized macrophages before incubation with particles is shown in Table 1.

Phagocytosis of particles has previously been reported to affect and reprogram the metabolism of phagocytosing cells [15], and the cellular content of lactate was chosen as an indicator of metabolic reprogramming, resulting in increased glycolysis [16]. Before the cells were lysed and samples were withdrawn for analysis, the adherent cells were washed with PBS to remove secreted lactate. To be able to evaluate the effect of Alhydrogel^®^ on lactate production, the effect of non-immune stimulating particles without any possibility of releasing Al ions was needed as a reference. Polystyrene (PS) beads with the same surface charge as Alhydrogel^®^, and having approximately the same size as the aggregates formed by Alhydrogel^®^, were chosen as reference particles.

**Figure 2:**
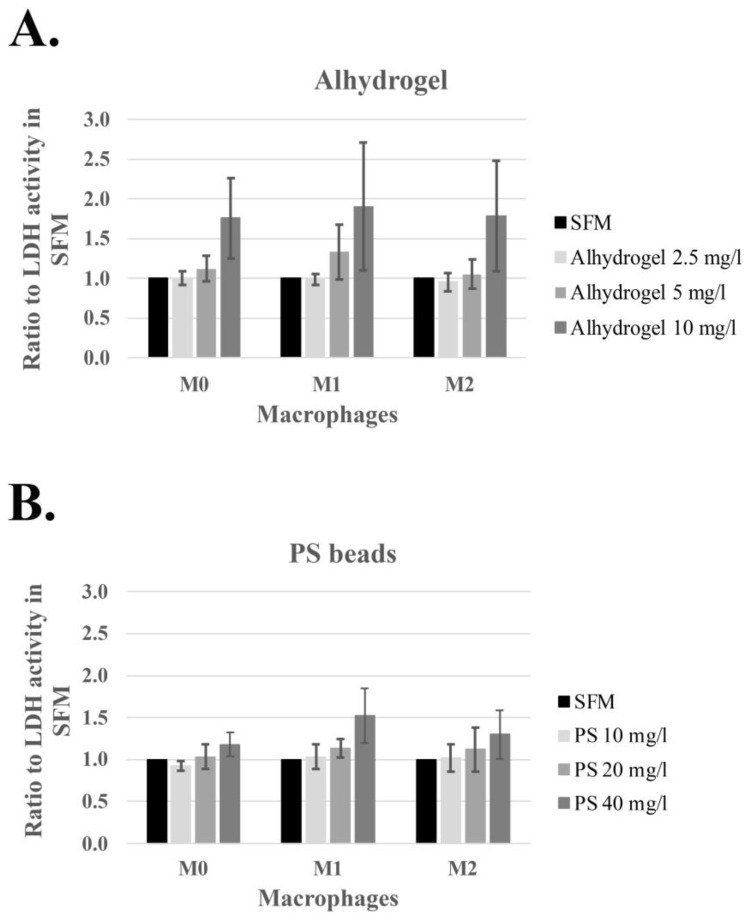
Increased LDH activity in culture medium from differentiated and polarized M0, M1, and M2 macrophages incubated for 24 h with Alhydrogel^®^ (**A**) and PS beads (**B**), average +/− standard deviation.

High particle loads are generally toxic to phagocytosing cells, and the severity can be dependent on the physico-chemical properties of the particles [17]; however, these properties must be investigated in biologically relevant conditions. The release of lactate dehydrogenase (LDH) from cells into the medium is a well-established sign of cell death [18]. Initially, the particle concentrations inducing toxicity were investigated by the release of LDH into the culture medium after incubation with Alhydrogel^®^ and PS beads during 24 h. The release of LDH into the medium was calculated relative to the LDH activity in the medium from cells incubated without any added particles (incubation with SFM). The average and standard deviation (SD) obtained from macrophages independently differentiated and polarized from seven (Alhydrogel^®^) and five (PS beads) different donors, respectively, are presented in Figure 2 (data from the individual donors are presented in the Supplementary Materials Table S1). Increased LDH release with a statistical *p*-value less than 0.05 was observed upon incubation with 10 µg/mL and 40 µg/mL of Alhydrogel^®^ and PS beads, respectively, indicating toxic effects by the particles at these concentrations. Drawing on these results, and thereby to avoid cell toxicity affecting the cellular lactate concentrations upon incubations with particles, the lactate production was investigated after incubations with lower concentrations than 10 µg/mL of Alhydrogel^®^ and 40 µg/mL of PS beads.

The lactate concentration was then adjusted by the results from the CellTiter assay to correspond to the lactate concentration found in the macrophage populations incubated in SFM without the addition of particles.

In total, macrophages were differentiated and polarized from nine different donors, and a large variation in the production of lactate by the differentiated and polarized macrophages not incubated with Alhydrogel^®^, i.e., the serum-free medium control of M0, M1, and M2 macrophages from each donor, was observed (Figure 2) and is summarized in Table 2 (data from the individual donors are presented in the Supplementary Materials Table S4).

**Figure 3:**
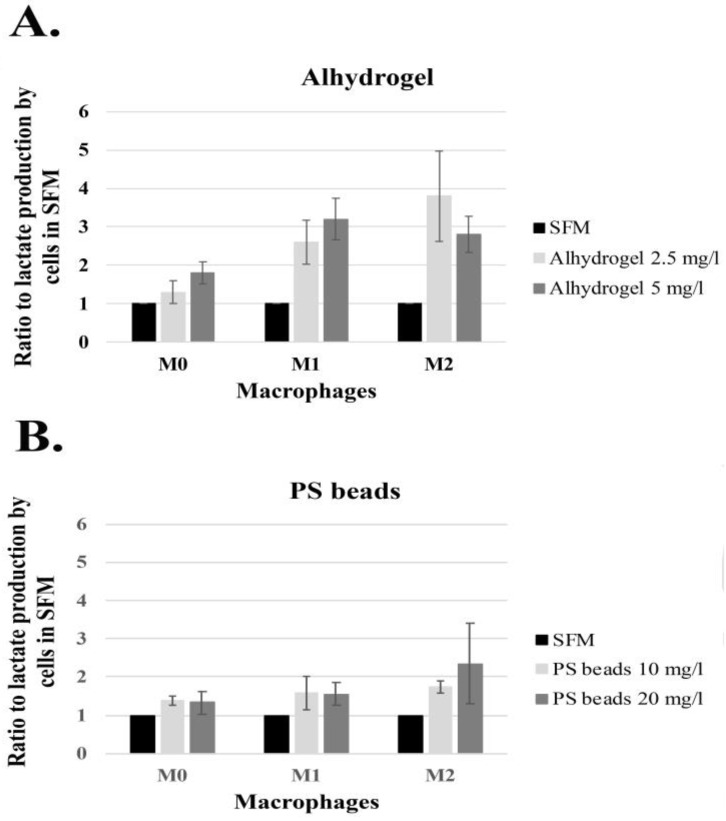
Normalized cellular lactate concentrations and standard error of the mean (SEM) from M0, M1, and M2 macrophages, average after incubation of Alhydrogel^®^ and macrophages independently differentiated and polarized from 9 donors (**A**), and respective PS beads from 6 donors, (**B**).

An almost 100% variation in the lactate concentration was observed from the untreated macrophages from the various donors (Table 2). To overcome the variation when investigating the effect of particles, the lactate concentrations in the serum-free medium control of macrophages from each individual donor were set as 1. The lactate concentrations after incubation with Alhydrogel^®^ and PS beads were then assessed relative to the SFM control after 24 h of incubation. This normalization process made it possible to assess the lactate production after incubation with Alhydrogel^®^ and PS beads. Average and normalized cellular lactate concentrations after incubations with Alhydrogel^®^ from nine donors and incubations with PS beads from six donors are presented in Figure 3. Tables showing the lactate concentrations and results from the CellTiter assay of differentiated and polarized macrophages from the individual donors can be found in the Supplementary Materials (Tables S2–S7).

Incubation with Alhydrogel^®^ resulted in increased lactate production in all three macrophage forms, and the increase caused by Alhydrogel^®^ was 2–4 times higher than the increase caused by PS beads based on the average lactate concentration in the cells. Regardless of normalization, the average lactate concentrations by the macrophages were still at variance (Figure 3) and to ensure that a significant increase in the cellular lactate content was at hand, a paired two tailed *t*-test was made using the cellular lactate concentrations after adjustment by the CellTiter assay (Supplementary Materials Tables S4 and S7). A significant difference in incubation with Alhydrogel^®^ was found for M0 as well as M1 and M2 macrophages (Table 3), although the highest significance level was observed for M1 macrophages with a *** significance level on incubation with 5 μg/mL. Additionally, regarding PS beads, only the M1 macrophages showed a statistically significant difference after incubation with 10 µg/mL, with a *p*-value of 0.013. Increased lactate production in either M0 or M2 macrophages could not be statistically verified.

## 3. Discussion

Based on the expression of CD markers and cytokine production (Table 1), the differentiated and polarized macrophages can be regarded as consistent models of quiescent M0 macrophages, inflammatory M1 macrophages, and alternatively activated M2 macrophages. All forms of macrophages phagocytose Alhydrogel^®^ result in a high intracellular load of precipitated aluminum salts [19]. The intracellular deposit of aluminum salt will most certainly affect the biochemical and biological processes within a cell.

To handle the intracellular ABA and to neutralize the effects of ABA and intracellular aluminum ions solubilized from the ABA, the energy demands of the cells are expected to increase. On incubation with Alhydrogel^®^, all forms of differentiated and polarized macrophages boosted the intra-cellular lactate content. The increased lactate production was statistically verified, and the highest significance level was found for the M1 macrophages. Unsurprisingly, increased lactate production was also observed for the M1 macrophages upon incubation with PS beads. Lactate is a molecular signature of glycolytic metabolism [16], and upon polarization to inflammatory M1 macrophages, the metabolism of the macrophages becomes reprogrammed to a glycolytic metabolism [12]. The activation of macrophages into inflammatory M1 macrophages results in a metabolic reprogramming of the cells. Generally, M1 macrophages exhibit increased glycolytic metabolism [12], whereas alternatively activated or anti-inflammatory M2 macrophages manifest enhanced oxidative phosphorylation [20]. Phagocytosis of particles increases the energy demand, and in reprogrammed inflammatory M1 macrophages, the energy will be obtained through glycolysis and hence an increased formation of lactate. However, it is worth noticing that phagocytosis of Alhydrogel^®^ resulted in a more pronounced production of lactate by the M1 macrophages compared to phagocytosis of PS beads.

More interesting is the increased lactate production induced by Alhydrogel^®^ on both M0 and M2 macrophages. This indicates a metabolic reprogramming of the quiescent M0 macrophages and of the polarized alternatively activated or anti-inflammatory M2 macrophages. Polarization influences the metabolic profile of macrophages [21]. Hypoxia-induced factors (HIFs) are major regulators of metabolism [22], and HIF-1α enhances aerobic glycolysis [22,23] by upregulating glycolytic enzymes. HIF-1α has also been reported to upregulate the expression of pro-inflammatory cytokines [12,24] and serves as a base in inducing trained immunity of macrophages [25], thus sustaining an inflammatory polarization of macrophages.

ABAs are not degradable by cells, and upon phagocytosis of ABAs, the cells will neither secrete nor degrade the ABA [19,26,27]. This will create a long-term accumulation of intracellular aggregates of aluminum salts and thereby a potential source of a constant concentration of aluminum ions in the cytosol [28]. An interesting aspect is whether ABAs affect and reprogram the cellular metabolism of the macrophages and thereby take part in the mechanism mediating the immune-stimulating properties of ABAs. Macrophages and dendritic cells are sentinel cells that are crucial initiators of inflammation that guide and direct the activation of the adaptive immune system. Metabolic reprogramming, inducing these cells into inflammatory and antigen-presenting cells (APCs), almost certainly results in the activation of the adaptive immune system. Upon phagocytosis, the ABAs are localized and accumulate in maturating phagosomes, and there are several possible mechanisms of their release into the cytosol [28]. ABAs released into the cytosol will become a long-lasting source of Al^3+^ ions with extensive effects on organelles and biological pathways. Aluminum ions have been reported to impact the TCA cycle, resulting in the accumulation of succinate [29], as well as the stabilization of HIF-1α [30]. Succinate and HIF-1α are both inducers of glycolytic metabolism, and phagocytosis of ABAs by quiescent M0 macrophages and by already polarized alternatively activated M2 macrophages may skew their metabolism, as shown by their increased lactate production upon incubation with Alhydrogel^®^ (Figure 3 and Table 3), toward a glycolytic metabolism. An increased glycolytic metabolism could then direct these macrophages and probably also immature and inactive DCs toward an inflammatory polarization at the same time as the ABAs mediate the uptake of antigens adsorbed on the adjuvant.

DCs and macrophages are cardinal cells in the induction of an immune response. Depletion of DCs and macrophages in mice has shown no reduction in the immune response upon immunization using aluminum adjuvants [7]. This indicates that the immune-stimulating mechanism by ABAs does not involve DCs and macrophages. However, DCs and macrophages are highly heterogenous populations with a considerable variation between infiltrating and resident macrophages. The lack of decreased immune response upon the depletion of phagocytosing cells can also be looked upon as being due to the plasticity of the sentinel cells acting as antigen-presenting cells, the limited number needed, or the fact that cells in a heterogenous population can replace each other on demand. Several mechanisms undoubtedly realize the immune-stimulating properties of ABAs, and the metabolic reprogramming of macrophages and possibly also of DCs could be one of several mechanisms governing the immune-stimulating properties of ABAs. ABAs are extensively used as adjuvants in vaccine formulations, and vaccines containing ABAs have a well-established track record [31] and are generally regarded as safe [32]. Vaccines containing ABAs are widely used and are sometimes considered to be used as a “gold standard” when assessing other adjuvants [33]. However, concerns regarding their use have been raised [34,35], and revealing the mechanism behind the immune-stimulating properties of ABAs is of ultimate interest in the effort of creating safer and more effective adjuvants.

## 4. Materials and Methods

### 4.1. Materials

Aluminum adjuvant preparations: Alhydrogel^®^ (AlO(OH)) was purchased from Brenntag Biosector (Frederikssund, Denmark), and aqueous suspension of amine-modified polystyrene, fluorescent yellow-green beads of 1 μm mean particle size was obtained from Sigma-Aldrich, St. Louis, MO, USA and referred to as PS-beads in the text. The cell culture medium used in all experiments was Gibco Macrophage-SFM serum-free medium, obtained from ThermoFisher Scientific Inc. and supplemented with 50 μg/mL gentamicin (Invitrogen AB, Göteborg, Sweden). Throughout the text, this medium is referred to as SFM medium. Human recombinant cytokines M-CSF, IL-4 and IFN-Ɣ of research grade came from Miltenyi Biotec (Bergisch Gladbach, Germany) and LPS, *E. coli* (serotype 0127:B8) from Sigma-Aldrich, St. Louis, MO, USA.

Macrophages were differentiated and polarized in 6-well and 96-well plates from VWR International, LLC, PA, USA.

### 4.2. Macrophage Differentiation and Polarization

Primary human macrophages were differentiated by a slight modification of the protocol described by Zarif et al. [36]. Briefly, peripheral blood mononuclear cells (PBMCs) from healthy donors were purchased from the local blood donation bank (Skånes universitetssjukus, SUS, Lund, Sweden) and separated by density centrifugation using Ficoll-Paque™ (GE Healthcare Life Sciences, Danderyd, Sweden). Highly purified classical monocytes were obtained with the Classical Monocytes MACS Isolation Kit (Miltenyi Biotec, Bergisch Gladbach, Germany), following the manufacturer’s instructions. After purification, classical monocytes were suspended in Macrophage-SFM at a concentration of 1 × 10^6^ cells/mL and seeded into 6- and 96-well plates using 300,000 cells/cm^2^ to adhere the monocytes. After 2 h at 37 °C in a humidified atmosphere with 5% CO_2_, the medium and non-adhered cells were removed and replaced with SFM containing 20 ng/mL M-CSF, 2 mL/well in the 6-well plates, and 200 µL/well in the 96-well plates. The cells were then incubated at 37 °C in a humidified atmosphere with 5% CO_2_ for 4 days, with the medium changed on day 2, to obtain quiescent M0 macrophages. To finally obtain polarized M1 and M2 macrophages, M0 cells were cultured for another 24 h in either an M1 cytokine cocktail (20 ng/mL M-CSF, 20 ng/mL IFN-γ and 20 ng/mL LPS) or an M2 cytokine cocktail (20 ng/mL M-CSF and 20 ng/mL IL-4). Quiescent M0 macrophages were cultured for another 24 h with 20 ng/mL M-CSF, and all cell treatments were performed in SFM.

### 4.3. Fluorescence Images of Differentiated and Polarized Macrophages

Monocytes were differentiated and polarized in culture chambers (BD Falcon CultureSlides, uncoated from BD Biosciences, San Jose, CA, USA) and incubated overnight with 2 µg/mL Lumogallion labelled Alhydrogel^®^ [37]. The macrophages were then washed with 2 × 400 μL PBS, fixed in 100 μL 1% (*w/v*) PFA for 15 min at room temperature, and washed again with 2 × 400 μL PBS. Finally, the cells were mounted using ProLong^^®^^ Gold Antifade Mountant with DAPI (Invitrogen AB, Sweden) and imaged using fluorescence microscopy (BX-53, Olympus LRI Instrument, AB). Controls consisted of differentiated and polarized macrophages incubated in SFM medium without any aluminum adjuvant.

### 4.4. Incubation with Alhydrogel^®^ and PS-Beads

The medium was removed from differentiated and polarized macrophages in 96-well plates (day 5 after initiation of differentiation/polarization) and 100 µL SFM medium containing 2.5 to 10 µg/mL of Alhydrogel^®^ (Al content was based on the concentration of aluminum in the Alhydrogel^®^, not on the molecular mass of AlO(OH)) or 5 to 40 µg/mL of PS beads. Each concentration of particles was performed in triplicate, and as reference, 100 µL of SFM medium without any particles was added. After 24 h at 37 °C in a humidified atmosphere with 5% CO_2_, the medium was collected, pooled, and centrifuged for 5 min at 5000× *g*, and the supernatant was collected and stored at −20 °C until assay of LDH activity. Adherent cells were washed with 100 µL PBS and then incubated for 30 min at 37 °C with 100 µL CellTiter reagent. After measurement of the fluorescence, inactivation solution was added to stop the cellular metabolization according to the manufacturer’s instructions for the lactate assay. Finally, the samples were neutralized and stored at −20 °C until assay of the lactate content.

The same number of monocytes were added to the wells when differentiation and polarization of macrophages were initiated. However, 6 days later, when the macrophages were incubated with particles, different numbers of cells were present in the wells. This will affect the outcome of the determined cellular lactate concentrations. To compensate for the cell number discrepancy, the CellTiter assay was used. The CellTiter assay measures protease activity within a cell and was used to estimate the cell numbers in the wells incubated with Alhydrogel^®^ and PS beads relative to the numbers of cells in wells incubated without any addition of particles, the serum-free medium control (SFM). The relative number of cells in the wells obtained from the CellTiter assay was then used to assign the lactate concentrations upon incubation with Alhydrogel^®^ and PS-beads corresponding to the same number of cells that were obtained in the control wells containing the equivalent form of macrophages incubated in the SFM medium.

Finally, as an attempt to compensate the large variation in lactate production between the donors from differentiated and polarized macrophages incubated in SFM medium without any addition of particles (Table 2), the cellular concentrations of lactate was normalized to the lactate concentrations obtained in the control macrophages from the individual donors, i.e., the corresponding forms of macrophages incubated in SFM.

### 4.5. LDH, CellTiter, and Lactate Assays

LDH activity was measured using CyQuant LDH Cytotoxicity Assay kit (Invitrogen AB, Sweden) according to the manufacturer’s instructions.

CellTiter-Fluor™ Cell Viability Assay kit (Promega Corporation, Madison, WI, USA) was used to determine the relative cell numbers from each cell treatment. The companys’ instructions were used to determine the relative cell number by measuring the fluorescence, excitation 390 nm, and emission 505 nm with a SpectraMax^^®^^ iD3 reader (Molecular Devices, San Jose, CA, USA).

Lactate determinations were performed according to the manufacturer’s instructions using Lactate- Glo™ Assay kit (Promega Corporation, Madison, WI, USA). The lactate concentrations were measured by recording the luminescence using a SpectraMax^^®^^ iD3 reader with an open wavelength setting and 1000 ms integration time. To obtain an optimal readout, white opaque optiPlate-96-well plates (PerkinElmer, Waltham, MA, USA) were used.

The lactate concentrations from the different treated cells were calculated using a lactate standard from 0–100 µM, and all samples were diluted to fit the standard.

### 4.6. Flow Cytometry

The medium was removed from differentiated and polarized macrophages in 6-well plates and washed with 1 mL PBS. Cells were eluted from the wells by adding 2 × 0.5 mL Accutase cell detachment solution (Invitrogen, ThermoFisher Scientific Inc., Waltham, MA, USA). Between each addition of Accutase, the cells were incubated for 5 min at room temperature, and the remaining cells were detached using a rubber police. Eluted cells were harvested and resuspended in PBS containing 0.1% BSA and 0.1% human IgG and stained with APC-labeled anti-CD80 (Miltenyi Biotec (Bergisch Gladbach, Germany) and PE-labeled anti CD206 (BD Bioscience, San Jose, CA, USA) and appropriately labeled isotype controls (R&D Systems, Minneapolis, MN, USA).

### 4.7. Cytokine ELISA

The human cytokines, IL-6 and TNF-α, were analyzed using DuoSet ELISA (R&D systems, Minneapolis, MN, USA), which was performed according to the manufacturer’s instructions.

### 4.8. Analyzing the Lactate Data

The CellTiter assay was used to estimate the number of cells from each treatment relative to the corresponding incubation in SFM medium without any addition of particles. These values were then used to convert the values of lactate concentrations obtained after incubations with Alhydrogel^®^ and PS-beads to what would have been obtained if the same number of cells were present in these wells as in the wells of cells treated without any particles. Converted lactate concentrations from the macrophages of the individual donors were then analyzed by a paired Student’s *t*-test using SPSS version 28.01.1 (14).

## 5. Conclusions

Most studies concerning the differentiation and polarization of macrophages are conducted in vitro. The differentiation and polarization of macrophages in vivo can be expected to be more complex, and the polarization from quiescent M0 macrophages into pro- or anti-inflammatory macrophages is probably not always clear-cut. It is hypothesized that during the activation of macrophages in vivo due to an infection, quiescent M0 macrophages are initially polarized with inflammatory M1 macrophages. After fulfilling their purpose, the macrophages switch to anti-inflammatory M2 macrophages to inhibit the inflammatory response and promote tissue repair [38]. A functional flexibility between the polarized forms of macrophages can be expected in vivo with the reassignment of M1 macrophages to phenotypical and functional M2 macrophages and vice versa, together with intermediates between the polarized forms of macrophages. In vitro incubation with Alhydrogel^®^ clearly induces increased production of lactate by not only inflammatory M1 macrophages but also quiescent M0 macrophages and polarized anti-inflammatory/alternatively activated M2 macrophages. M0 and M2 macrophages are assumed to use oxidative phosphorylation as the main energy supply, and increased lactate production indicates a metabolic reprogramming of these macrophages. A consequence of exposure to ABAs such as Alhydrogel^®^ most likely is a potential intracellular depot of aluminum ions that may induce or promote metabolic reprogramming of the macrophages and thereby skew the polarization, promoting M0 and M2 macrophages toward inflammatory and antigen-presenting macrophages. In vivo, this may increase the number of antigen-presenting cells generated from macrophages and DCs, and it is hypothesized that the increase is a part of the mechanism providing the immune-stimulating properties of ABAs.

## Figures and Tables

**Table 1:** Expression of CD markers and cytokine production by in vitro differentiated and polarized macrophages.

Macrophages	CD Marker (MFI ^a^ × 10^3^)		Cytokines ^b^	
	CD80	CD206	TNF-α	IL-6
M0	2.7–8.2	43.0–53.7	31 pg/mL	9 pg/mL
	−/+	++	or lower	or lower
M1	12.2–51.4	21.8–41.4	500 pg/mL	600 pg/mL
	++	+	or higher	or higher
M2	4.0–12.4	135–267	31 pg/mL	9 pg/mL
	−/+	+++	or lower	or lower

The intensity of the CD markers is shown by: negative (−), low intensity (+), medium intensity (++) and high intensity (+++). ^a^ Mean Fluorescence Intensity. ^b^ Concentration range of the standard curve used in the ELISAs; TNF-α: 31 pg/mL to 1000 pg/mL, and IL-6: 9 pg/mL to 600 pg/mL.

**Table 2:** Average lactate production +/− standard deviations by differentiated and polarized macrophages not incubated with Alhydrogel^®^, i.e., the serum-free medium control of M0, M1, and M2 macrophages, independently differentiated and polarized from nine donors.

Cells	Average Lactate Concentrations with Standard Deviations (μM)
M0 macrophages	27.9 +/− 23.7
M1 macrophages	24.2 +/− 21.3
M2 macrophages	30.9 +/− 33.1

**Table 3:** Statistic evaluation of cellular lactate content after incubation with Alhydrogel^®^ and PS beads. A paired two-tailed student’s *t*-test was used to calculate the *p*-values. *p*-values < 0.05 (*), <0.01 (**), and <0.001 (***) were considered significant.

Cells	Alhydrogel^®^ (*p*-Value)		PS Beads (*p*-Value)	
	2.5 μg/mL	5 μg/mL	10 μg/mL	20 μg/mL
M0 macrophages	0.084	0.003 (**)	0.161	0.415
M1 macrophages	0.005 (**)	0.001 (***)	0.013 (*)	0.959
M2 macrophages	0.061	0.020 (*)	0.087	0.122

## Data Availability

Additional data and files have been uploaded as Supplementary Files.

## Supplementary Materials

The following supporting information can be downloaded at: https://www.mdpi.com/article/10.3390/ijms24054409/s1.
