# Supplementary material for: Metabolic Reprogramming of Macrophages upon In Vitro Incubation with Aluminum-Based Adjuvant
Source: Int J Mol Sci. 2023 Feb 23;24(5):4409. doi: 10.3390/ijms24054409 (PMC10002480; doi:10.3390/ijms24054409)
Supplement: Supplementary file 1 [file ijms-24-04409-s001.zip › ijms-2154237-supplementary.pdf]

Table S1. Relative lactate dehydrogenase (LDH) activity in conditioned culture medium from M0, M1 and M2 macrophages of the individual donors after incubation with increasing concentrations of Alhydrogel® and PS beads. SFM stands for medium control.

Incubation with Alhydrogel®

**M0**	**Donor**						
Conc Alhydrogel (µg/ml)	1	2	3	4	5	6	7
0 (SFM)	1.00	1.00	1.00	1.00	1.00	1.00	1.00
2.5	0.90	1.04	1.10	0.95	1.12	0.93	0.97
5	0.96	1.35	1.09	1.00	1.34	1.09	1.01
10	1.70	2.34	1.61	1.03	2.12	-	-
**M1**	**Donor**						
Conc Alhydrogel (µg/ml)	1	2	3	4	5	6	7
0 (SFM)	1.00	1.00	1.00	1.00	1.00	1.00	1.00
2.5	0.93	1.05	1.02	0.96	0.88	1.05	1.03
5	1.49	1.99	1.19	1.16	0.86	1.27	1.36
10	2.59	2.91	1.69	1.12	1.22	-	-
**M2**	**Donor**						
Conc Alhydrogel (µg/ml)	1	2	3	4	5	6	7
0 (SFM)	1.00	1.00	1.00	1.00	1.00	1.00	1.00
2.5	0.74	0.94	0.96	0.91	1.10	1.05	0.99
5	0.76	1.11	1.01	0.90	1.21	1.29	1.10
10	1.39	2.74	1.59	0.98	2.21	-	-

Incubation with PS beads

**M0**	**Donor**				
Conc PS beads (µg/ml)	1	2	3	4	5
0 (SFM)	1.00	1.00	1.00	1.00	1.00
10	0.83	0.99	0.92	0.92	0.95
20	0.92	1.22	0.93	0.92	1.17
40	-	1.28	0.97	1.27	1.20
**M1**	**Donor**				
Conc PS beads (µg/ml)	1	2	3	4	5
0 (SFM)	1.00	1.00	1.00	1.00	1.00
10	0.89	1.06	1.10	0.88	1.23
20	1.03	1.32	1.12	1.07	1.13
40		1.99	1.26	1.41	1.43
**M2**	**Donor**				
Conc PS beads (µg/ml)	1	2	3	4	5
0 (SFM)	1.00	1.00	1.00	1.00	1.00
10	0.90	0.97	1.01	0.90	1.30
20	0.88	1.27	1.01	0.93	1.51
40	-	1.22	0.92	1.48	1.57

Table S2. Lactate concentrations (µM) of M0, M1 and M2 macrophages from donors 1-9 after incubation with increasing concentrations of Alhydrogel®. SFM stands for medium control.

**M0**	**Donor 1-9** (lactate (µM))								
Conc Alhydrogel (µg/ml)	1	2	3	4	5	6	7	8	9
0 (SFM)	39.8	29.7	10.0	80.1	26.2	13.0	39.3	5.2	7.5
2.5	33.2	29.2	13.8	114.9	41.2	14.5	72.2	0.9	8.3
5	55.7	39.9	36.5	89.6	31.4	18.8	57.1	1.8	6.7

**M1**	**Donor 1-9** (lactate (µM))								
Conc Alhydrogel (µg/ml)	1	2	3	4	5	6	7	8	9
0 (SFM)	23.1	20.0	7.6	74.3	24.9	16.8	37.0	3.7	10.2
2.5	43.1	29.0	18.7	99.6	23.1	13.2	39.7	9.9	24.0
5	54.1	40.7	40.0	90.6	35.6	23.6	71.1	5.5	14.2

**M2**	**Donor 1-9** (lactate (µM))								
Conc Alhydrogel (µg/ml)	1	2	3	4	5	6	7	8	9
0 (SFM)	41.4	15.7	8.7	98.2	25.4	7.7	70.2	3.2	7.5
2.5	44.0	22.8	62.7	130.5	141.3	11.2	122.9	4.4	15.2
5	89.1	27.1	50.3	100.8	38.7	21.0	80.6	2.9	13.9

Table S3. Relative CellTiter data of M0, M1 and M2 macrophages obtained from donors 1-9 after incubation with increasing concentrations of Alhydrogel®. SFM stands for medium control.

**M0**	**Donor 1-9**								
Conc Alhydrogel (µg/ml)	1	2	3	4	5	6	7	8	9
0 (SFM)	1.00	1.00	1.00	1.00	1.00	1.00	1.00	1.00	1.00
2.5	0.98	0.93	0.98	0.92	0.48	1.02	0.91	0.36	0.79
5	0.80	0.79	1.04	0.96	0.46	0.94	0.79	0.26	0.68

**M1**	**Donor 1-9**								
Conc Alhydrogel (µg/ml)	1	2	3	4	5	6	7	8	9
0 (SFM)	1.00	1.00	1.00	1.00	1.00	1.00	1.00	1.00	1.00
2.5	0.79	0.75	0.96	0.77	0.43	0.94	0.94	0.43	0.61
5	0.72	0.68	0.87	0.86	0.40	1.19	0.92	0.28	0.50

**M2**	**Donor 1-9**								
Conc Alhydrogel (µg/ml)	1	2	3	4	5	6	7	8	9
0 (SFM)	1.00	1.00	1.00	1.00	1.00	1.00	1.00	1.00	1.00
2.5	0.71	0.68	0.96	0.76	0.47	0.90	0.83	0.37	0.56
5	0.61	0.72	1.04	1.02	0.43	0.86	0.92	0.32	0.55

Table S4. Corrected lactate concentration (μM) of M0, M1 and M2 macrophages from donors 1-9 after incubation with increasing concentrations of Alhydrogel®. SFM stands for medium control.

**M0**	**Donor 1-9** (lactate (μM))								
Conc Alhydrogel (μg/ml)	1	2	3	4	5	6	7	8	9
0 (SFM)	39.8	29.7	10.0	80.1	26.2	13.0	39.3	5.2	7.5
2.5	33.9	31.4	14.1	124.9	85.8	14.2	79.3	2.5	10.5
5	69.6	50.5	35.1	93.3	68.3	20.0	72.3	6.9	9.9

**M1**	**Donor 1-9** (lactate (μM))								
Conc Alhydrogel (μg/ml)	1	2	3	4	5	6	7	8	9
0 (SFM)	23.1	20.0	7.6	74.3	24.9	16.8	37.0	3.7	10.2
2.5	54.6	38.7	19.5	129.4	53.7	14.0	42.2	23.0	39.3
5	75.1	59.9	46.0	105.3	89.0	19.8	77.3	19.6	28.4

**M2**	**Donor 1-9** (lactate (μM))								
Conc Alhydrogel (μg/ml)	1	2	3	4	5	6	7	8	9
0 (SFM)	41.4	15.7	8.7	98.2	25.4	7.7	70.2	3.2	7.5
2.5	62.0	33.5	65.3	171.7	300.6	12.4	148.1	11.9	27.1
5	146.1	37.6	48.4	98.8	90.0	24.4	87.6	9.1	25.3

Table S5. Lactate concentrations (μM) of M0, M1 and M2 macrophages from donors 1-6 after incubation with increasing concentrations of PS-beads. SFM stands for medium control.

**M0**	**Donor 1-6** (lactate (μM))					
Conc PS-beads (μg/ml)	1	2	3	4	5	6
0 (SFM)	39.3	26.5	5.2	7.5	6.1	2.0
10	65.7	34.2	1.3	4.5	9.5	3.0
20		16.5	0.5	8.0	6.2	4.3

**M1**	**Donor 1-6** (lactate (μM))					
Conc PS-beads (μg/ml)	1	2	3	4	5	6
0 (SFM)	37.0	42.3	3.7	10.2	6.4	2.2
10	41.9	37.6	2.0	7.8	10.2	3.3
20		13.5	1.7	12.0	8.0	3.3

**M2**	**Donor 1-6** (lactate (μM))					
Conc PS-beads (μg/ml)	1	2	3	4	5	6
0 (SFM)	70.2	30.2	3.2	7.5	5.9	2.6
10	92.8	58.6	2.1	8.4	7.3	3.4
20		40.3	3.7	11.5	6.3	2.5

Table S6. Relative CellTiter data of M0, M1 and M2 macrophages obtained from donors 1-6 after incubation with increasing concentrations of PS-beads. SFM stands for medium control.

**M0**	**Donor 1-6**					
Conc PS-beads (µg/ml)	1	2	3	4	5	6
0 (SFM)	1.00	1.00	1.00	1.00	1.00	1.00
10	0.98	0.86	0.31	0.53	1.11	0.90
20		0.60	0.23	0.51	1.04	1.00

**M1**	**Donor 1-6**					
Conc PS-beads (µg/ml)	1	2	3	4	5	6
0 (SFM)	1.00	1.00	1.00	1.00	1.00	1.00
10	1.04	0.90	0.30	0.50	0.92	0.65
20		0.72	0.27	0.48	0.86	0.89

**M2**	**Donor 1-6**					
Conc PS-beads (µg/ml)	1	2	3	4	5	6
0 (SFM)	1.00	1.00	1.00	1.00	1.00	1.00
10	0.94	0.91	0.31	0.55	1.11	0.77
20		0.62	0.30	0.41	0.94	1.14

Table S7. Corrected lactate concentration (μM) of M0, M1 and M2 macrophages from donors 1-6 after incubation with increasing concentrations of PS-beads. SFM stands for medium control.

**M0**	**Donor 1-6 (lactate (μM))**					
Conc PS-beads (μg/ml)	1	2	3	4	5	6
0 (SFM)	39.3	26.5	5.2	7.5	6.1	2.0
10	67.0	39.8	4.2	8.5	8.6	3.3
20		27.5	2.2	15.7	6.0	4.3

**M1**	**Donor 1-6 (lactate (μM))**					
Conc PS-beads (μg/ml)	1	2	3	4	5	6
0 (SFM)	37.0	42.3	3.7	10.2	6.4	2.2
10	40.3	6.3	6.7	15.6	11.1	5.1
20		18.8	6.3	25.0	9.3	3.7

**M2**	**Donor 1-6 (lactate (μM))**					
Conc PS-beads (μg/ml)	1	2	3	4	5	6
0 (SFM)	70.2	30.2	3.2	7.5	5.9	2.6
10	98.7	64.4	6.8	15.3	6.6	4.4
20		65.0	12.3	28.0	6.7	2.2
